# Investigation of Anticancer Properties of Monoterpene-Aminopyrimidine Hybrids on A2780 Ovarian Cancer Cells

**DOI:** 10.3390/ijms241310581

**Published:** 2023-06-24

**Authors:** Viktória Nagy, Raji Mounir, Gábor J. Szebeni, Zsolt Szakonyi, Nikolett Gémes, Renáta Minorics, Péter Germán, István Zupkó

**Affiliations:** 1 Institute of Pharmacodynamics and Biopharmacy, University of Szeged, H-6720 Szeged, Hungary; nagy.viktoria07@gmail.com (V.N.);; 2 Institute of Pharmaceutical Chemistry, University of Szeged, H-6720 Szeged, Hungary; 3 Laboratory of Functional Genomics, Eötvös Loránd Research Network Biological Research Centre, Institute of Genetics, H-6726 Szeged, Hungary; 4 Interdisciplinary Centre of Natural Products, University of Szeged, H-6720 Szeged, Hungary

**Keywords:** monoterpene, aminoalcohol, antiproliferative, G2/M arrest, antimetastatic, ovarian cancer

## Abstract

The present study aimed to characterize the antiproliferative and antimetastatic properties of two recently synthesized monoterpene-aminopyrimidine hybrids (**1** and **2**) on A2780 ovary cancer cells. Both agents exerted a more pronounced cell growth inhibitory action than the reference agent cisplatin, as determined by the MTT assay. Tumor selectivity was assessed using non-cancerous fibroblast cells. Hybrids **1** and **2** induced changes in cell morphology and membrane integrity in A2780 cells, as evidenced by Hoechst 33258–propidium iodide fluorescent staining. Cell cycle analysis by flow cytometry revealed substantial changes in the distribution of A2780 ovarian cancer cells, with an increased rate in the subG1 and G2/M phases, at the expense of the G1 cell population. Moreover, the tested molecules accelerated tubulin polymerization in a cell-free in vitro system. The antimetastatic properties of both tested compounds were investigated by wound healing and Boyden chamber assays after 24 and 48 h of incubation. Treatment with **1** and **2** resulted in time- and concentration-dependent inhibition of migration and invasion of A2780 cancer cells. These results support that the tested agents may be worth of further investigation as promising anticancer drug candidates.

## 1. Introduction

According to estimates from the World Health Organization (WHO), cancer ranks as one of the major health burdens and a leading cause of mortality worldwide. Therefore, it can be concluded that malignancies are the leading cause of premature death in developed countries. Based on the latest Global Cancer Observatory (GLOBOCAN) survey, 19.3 million new cancer cases, and almost ten million cancer-related deaths were registered in 2020. Several factors, including aging and population growth, as well as the prevalence and distribution of the main risk factors for cancer can lead to such a high cancer incidence and mortality, rapidly growing globally [1,2].

Among females, gynecological malignancies are the most commonly diagnosed tumor types and the leading causes of cancer death. Accordingly, ovarian cancer is the most lethal female reproductive cancer, associated with the highest mortality rate among gynecological tumors. In most cases, it is diagnosed at an advanced stage, when the cancer already spread to other tissues [3].

Several risks factors for the development of ovarian cancer were identified, including family history of ovarian cancer in a first-degree relative, genetic predisposition (e.g., mutations of *BRCA1* and *BRCA2* genes), hereditary nonpolyposis colorectal cancer, endometriosis, postmenopausal hormone therapy, and lifestyle effects, for instance, obesity and smoking [4].

The lack of specific symptoms and adequate screening can lead to delayed diagnosis in advanced stages with high dissemination of the disease, all contributing to poor prognosis [5,6]. Correspondingly, novel treatment options, including innovative drugs with improved efficacy profiles, are eagerly required.

The research for and development of novel oncology agents posed significant challenges for all aspects of chemistry and pharmacology since the early 1940s [7]. Initially, most of the materials screened were pure compounds of synthetic origin; however, it was recognized that natural products are an excellent source of complex chemical structures possessing a wide variety of biological activities [8]. As a result, many natural products were introduced into clinical trials and clinical practice, and were used as lead or model molecules for structure optimization, as well as for developing safer and more tolerable drugs [9].

The best-known of currently used anticancer drugs are *Vinca* alkaloids vinblastine and vincristine. These agents can be isolated from endophytic fungi associated with the source plant. They act via tubulin polymerization, and are utilized in combination with other anticancer agents in various cancer types (leukemia, advanced testicular cancer, breast cancer, etc.). They exert their antitumor effect via binding microtubular proteins, which leads to disturbed tubulin polymerization and disrupts microtubule assembly during mitosis, as well as induces the metaphase arrest of the cell cycle [10]. Another natural anticancer agent, paclitaxel, was isolated from the bark of Pacific yew (*Taxus brevifolia*), and also occurs in the leaves of various *Taxus* species. It is also widely utilized in oncology, to treat, for example, breast, ovarian, and non-small cell lung cancer, etc. By stabilizing the microtubule polymer and preventing microtubules from disassembly, paclitaxel arrests the cell cycle in the G0/G1 and G2/M phases and, thus, induces cell death [11]. Several binding sites were identified on the surface of the dimer of α- and β-tubulin. These include the vinca site binding *Vinca* alkaloids, colchicine site binding colchicine, combretastatins, and 2-methoxyestradiol and taxane site binding taxanes and epothilones [12].

Although natural anticancer agents can be potent, they tend to have poor pharmacokinetic properties; thus, chemical derivatization is required to obtain novel analogs suitable for further drug development [8].

The significance of natural products in health care is supported by a report claiming that over 60% of current anticancer agents in clinical use are natural products, their derivatives or analogs, and 74% of the essential drugs consist of plant-derived active ingredients [10].

Terpenoids are the largest class of natural products, and represent an important class of secondary plant metabolites. They are composed of five carbon isoprene units, and they are divided into subclasses based on their structures [11]. They can target several stages of cancer development, including cell proliferation, cell death, angiogenesis, and metastasis formation [13]. The anticancer effect of several terpenoids is mediated by targeting nuclear factor kB (NF-kB), Janus kinases-signal transducer and activated transcription proteins (JAK-STAT), activator protein-1 (AP-1), metalloproteinases (MMPs) and DNA topoisomerase I and II, or the relevant compounds may block the endoplasmic reticulum (ER) the calcium ATPase pump, inhibit the proteasome, activate p53, and modulate DNA minor grooves [14]. Terpenoids were reported to induce cell death by a particular form of apoptosis called autophagy. It is a self-digesting process characterized by forming double-membrane vacuoles and autophagosomes, followed by merging with lysosomes. These processes can lead to the degradation of molecules; thus, organelles become dysfunctional [15].

**Figure 1:**
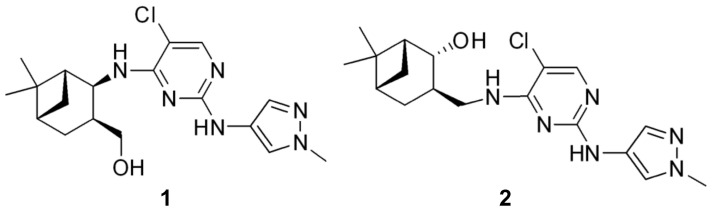
Chemical structures of the investigated monoterpene-based diaminopyrimidine analogs.

Monoterpenes exhibit a wide variety of biological activities, including anti-inflammatory, antibacterial, and antioxidant actions. Numerous scientific works explored the bioactivity of monoterpenes, including their anticancer potential in particular, providing in vitro evidence of efficacy against several cancer cell lines [13]. In a previous study, we reported on synthesizing a series of monoterpene-based diaminopyrimidine analogs, and we successfully demonstrated their antiproliferative properties in vitro on a panel of human adherent cancer cell lines [16]. Two of the prepared and tested analogs, **1** and **2** (Figure 1), exhibited outstanding activity. Our current study aimed to assess the antitumor and antimetastatic properties of these potent analogs, as well as to elucidate their mechanism of action.

## 2. Results

### 2.1. Antiproliferative Activities of the Test Compounds

Our choice for the particular compounds to be tested was based on previously established structure-activity relationship, as well as on the evidence for the antiproliferative properties (percentage growth inhibition) of the synthetized monoterpene-based diaminopyrimidine analogs [16]. Those investigated in the current study (compounds **1** and **2**) were found to exhibit a remarkable antiproliferative effect against A2780 cells (calculated IC_50_ values: 0.77 µM and 2.86 µM, respectively; Table 1). The obtained IC_50_ values were comparable to that of the reference agent cisplatin. Non-cancerous fibroblast cells (NIH/3T3) were also utilized to characterize tumor selectivity (TS) of the test substances. Based on these results, our compounds are selective towards ovarian carcinoma cells. Therefore, the A2780 cell line was selected for further in vitro investigations aimed at elucidating the mechanism of action of **1** and **2**.

### 2.2. Effects of the Test Compounds on Cell Morphology

**Figure 2:**
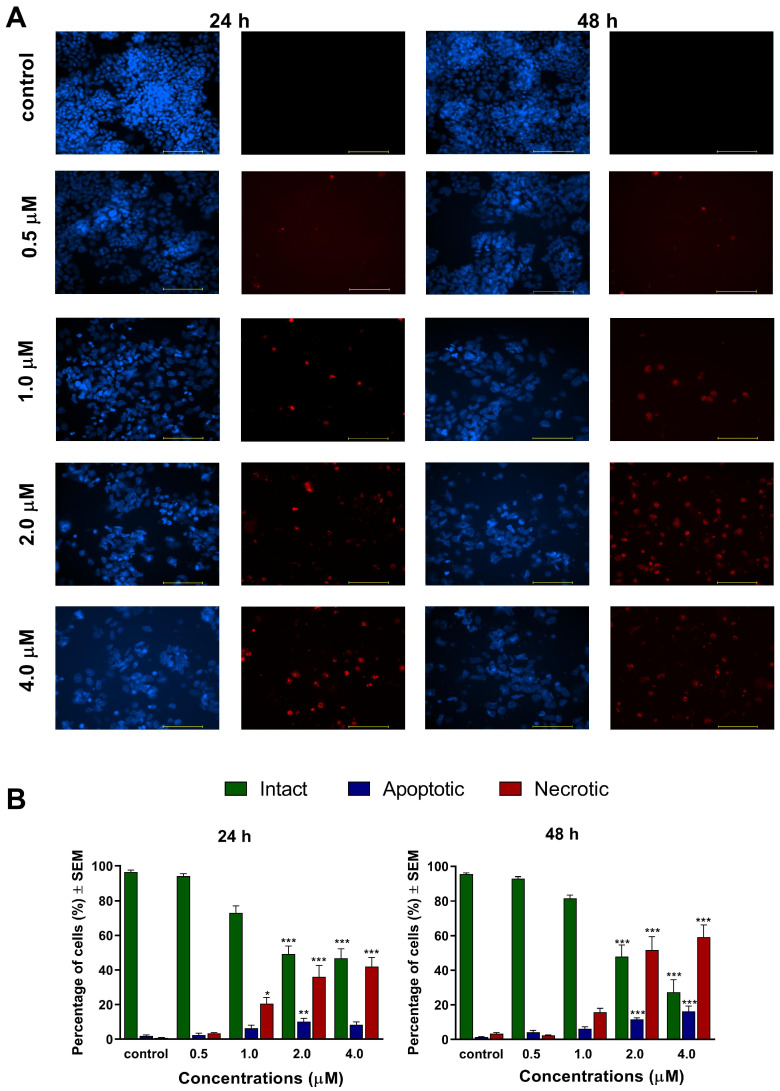
Morphological changes of A2780 cells after 24 h and 48 h of exposure to compound **1**, visualized by HOPI double staining. (**A**): Representative image pairs of the same field; blue fluorescence (**left pictures**) indicates Hoechst 33258, and red fluorescence (**right panels**) shows propidium accumulation. The bar in the pictures indicates 100 μm. (**B**): Percentages of intact, apoptotic, and necrotic cell populations. *, ** and *** indicate significance at *p* < 0.05, *p* < 0.01, and *p* < 0.001, respectively, compared to control. Data are from three independent experiments performed in triplicate, non-significant changes are not shown.

**Figure 3:**
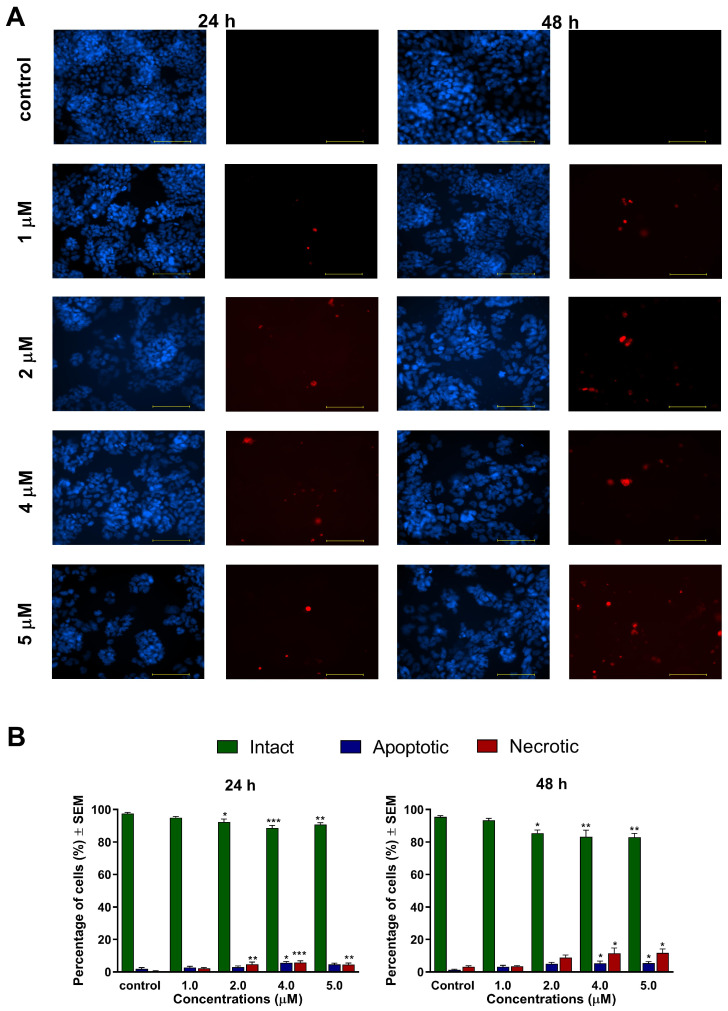
Morphological changes of A2780 cells after 24 h and 48 h of exposure to compound **2**, visualized by HOPI double staining. (**A**): Representative image pairs of the same field; blue fluorescence (**left pictures**) indicates Hoechst 33258, and red fluorescence (**right panels**) shows propidium accumulation. The bar in the pictures indicates 100 μm. (**B**): Percentages of intact, apoptotic, and necrotic cell populations. *, ** and *** indicate significance at *p* < 0.05, *p* < 0.01, and *p* < 0.001, respectively, compared to control. Data are from three independent experiments performed in triplicate, non-significant changes are not shown.

To characterize the changes in cell morphology and cell membrane integrity of ovarian cancer cells induced by the test compounds, fluorescent nuclei staining was performed using Hoechst 33,258 and propidium iodide dyes after 24 h and 48 h of exposure to 0.5, 1, 2, or 4 μM of **1**, and 1, 2, 4, or 5 μM of **2**. Images of the same field were taken using different optical blocks. For both compounds, our results generally revealed a concentration-dependently elevated ratio of apoptotic and necrotic cells after 48 h, accompanied by a significant decline in the ratio of viable intact cells (Figure 2 and Figure 3). Compound **1** induced a more pronounced apoptotic or necrotic damage, which can be regarded as a sign of increased potency compared to compound **2**. Representative microscopic images of higher resolution are available in the Supplementary Materials (Figures S1–S5).

### 2.3. Effects of the Test Compounds on Cell Cycle

**Figure 4:**
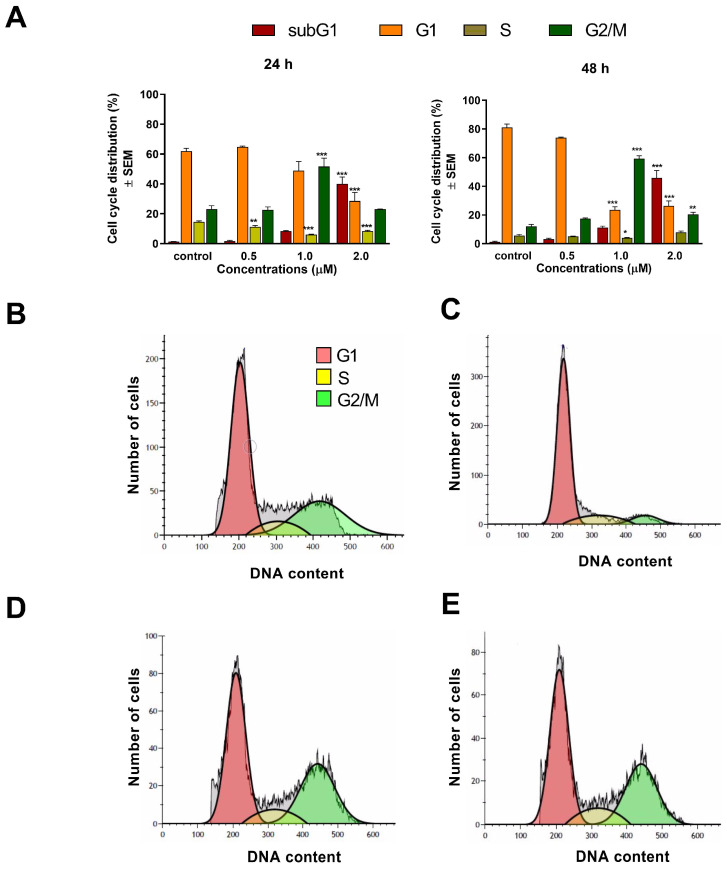
Effect of compound **1** on cell cycle distribution of A2780 cells treated with 0.5, 1, and 2 μM of the test substance for 24 and 48 h (**A**). *, **, and *** indicate significance at *p* < 0.05, *p* < 0.01, and *p* < 0.001, respectively, compared to control. Data are from three independent experiments performed in triplicate, non-significant changes are not shown. Representative histograms: control after 24 h (**B**), control after 48 h (**C**), 2 μM compound **1**, incubated for 24 h (**D**), and 2 μM compound **1**, incubated for 48 h (**E**).

**Figure 5:**
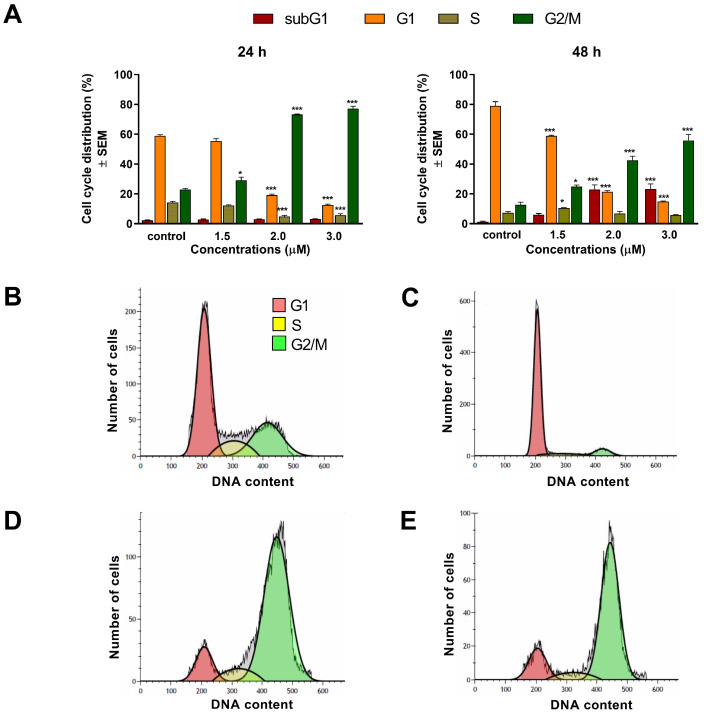
Effect of compound **2** on cell cycle distribution of A2780 cells treated with 1.5, 2, and 3 μM of the test substance for 24 and 48 h (**A**). * and *** indicate significance at *p* < 0.05 and *p* < 0.001, respectively, compared to control. Data are from three independent experiments performed in triplicate, non-significant changes are not shown. Representative histograms: control after 24 h (**B**), control after 48 h (**C**), 3 μM compound **2**, incubated for 24 h (**D**), and 3 μM compound **2**, incubated for 48 h (**E**).

The effects of our test compounds on cell cycle were investigated by flow cytometry after 24 h and 48 h of incubation. The applied concentrations were based on calculated IC_50_ values, and the ratio of cell populations in various cell cycle phases (subG1, G1, S, and G2/M) were determined. A concentration-dependent rise of hypodiploid subG1 cell populations was regarded as a marker of induced apoptosis. Treatment with 1 μM of compound **1** elicited a significant accumulation of cells in the G2/M phase and a reduction in the proportion of cells in the S phase after 24 h of incubation. Higher concentrations resulted in serious cell cycle disturbances, dominated by subG1 accumulation. After 48 h of incubation, these changes became even more pronounced (Figure 4). Treatment with compound **2** for 24 h significantly and dose-dependently decreased the rate of cells in the G1 phase, accompanied by a substantial elevation of the G2/M cell population after 24 h of incubation (Figure 5). More prolonged incubation (48 h) resulted in a gradual accumulation of hypodiploid cells. These results suggest that **1** and **2** induce marked cell cycle arrest in the G2/M phase in a dose-dependent manner.

### 2.4. Effects of the Test Compounds on Tubulin Polymerization

**Figure 6:**
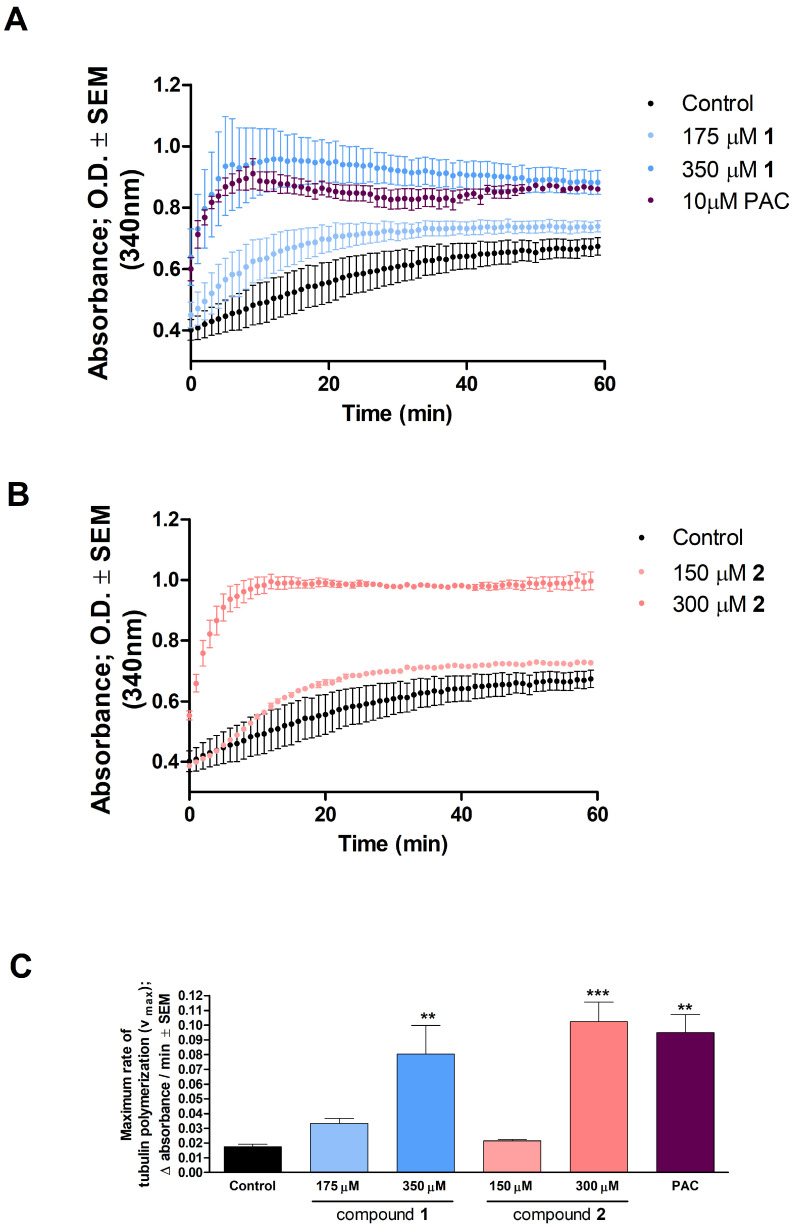
Compounds **1** and **2** affect tubulin polymerization ((**A**,**B**), respectively). Paclitaxel (PAC) was included as a reference agent. (**C**): calculated maximum values for the rate of tubulin polymerization. ** and *** indicate significance at *p* < 0.01 and *p* < 0.001, respectively, compared to control. Findings are based on the results of two independent experiments, both performed in duplicate. Non-significant changes are not shown.

A cell-free photometric assay was used to determine the direct effects of the test compounds on microtubule formation. The relatively high concentrations (175–350 and 150–300 μM for **1** and **2**, respectively) applied are based on the calculated IC_50_ values, in accordance with the manufacturer’s recommendation. Compared with untreated control samples, the highest concentrations of **1** and **2** induced a significant acceleration of tubulin polymerization. In addition, a considerable rise in the *V*max value was recorded at 350 μM of **1** and 300 μM of **2** compared to control (Figure 6).

### 2.5. Antimigratory Activities of the Test Compounds

**Figure 7:**
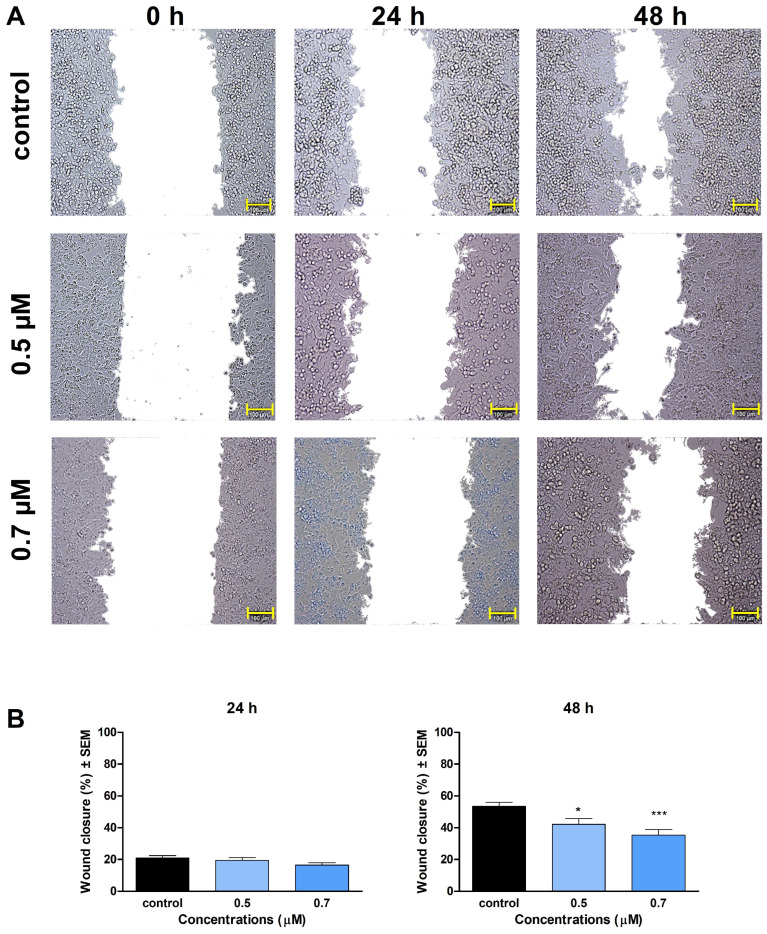

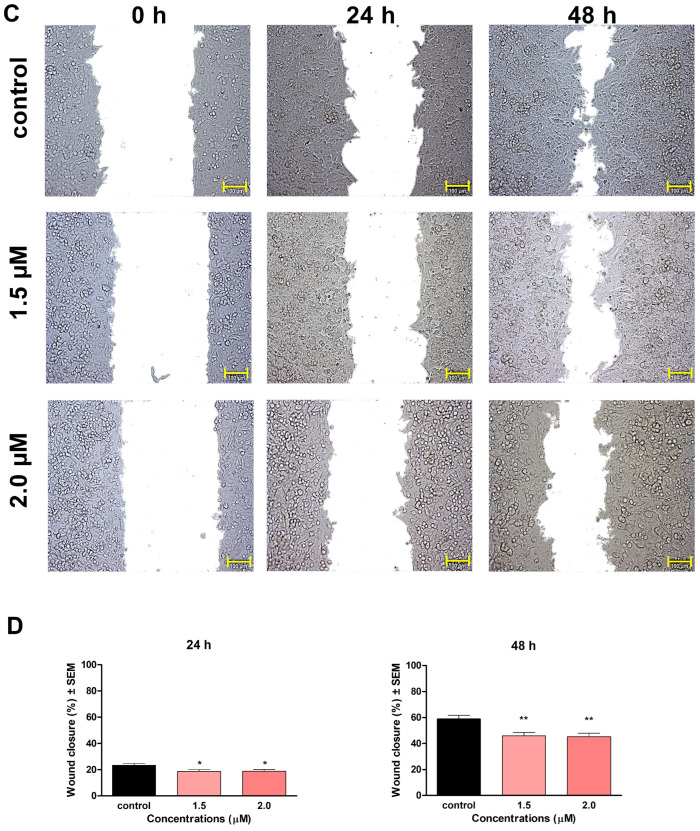
Effect of compounds **1** and **2** on migration of A2780 cells. (**A**): representative images taken at 24 and 48 h post-treatment with compound **1**. (**B**): calculated wound closure values determined after treatment with **1**. (**C**): representative images taken at 24 and 48 h post-treatment with compound **2**. (**D**): calculated wound closure values determined after treatment with **2**. The bar in the images indicates 100 μm. *, **, and *** indicate significance at *p* < 0.05, *p* < 0.01, and *p* < 0.001, respectively. Findings are based on the results of 4 independent experiments, all performed in triplicate. Non-significant changes are not shown.

The antimigratory properties of **1** and **2** were characterized in a wound-healing assay, via determining the cell-free area at 0, 24, and 48 h post-treatment, using samples with reduced serum-containing medium. Treatment with compound **1** at a concentration of 0.5 or 0.7 μM elicited no substantial effects after 24 h of incubation. However, a significant inhibition of cell migration was detected after 48 h at both concentrations (Figure 7). On the other hand, statistically significant inhibition of cell migration was evident 24 and 48 h post-treatment for 1.5 µM and 2.0 µM of compound **2,** compared to the control.

### 2.6. Anti-Invasion Effects of the Test Compounds

**Figure 8:**
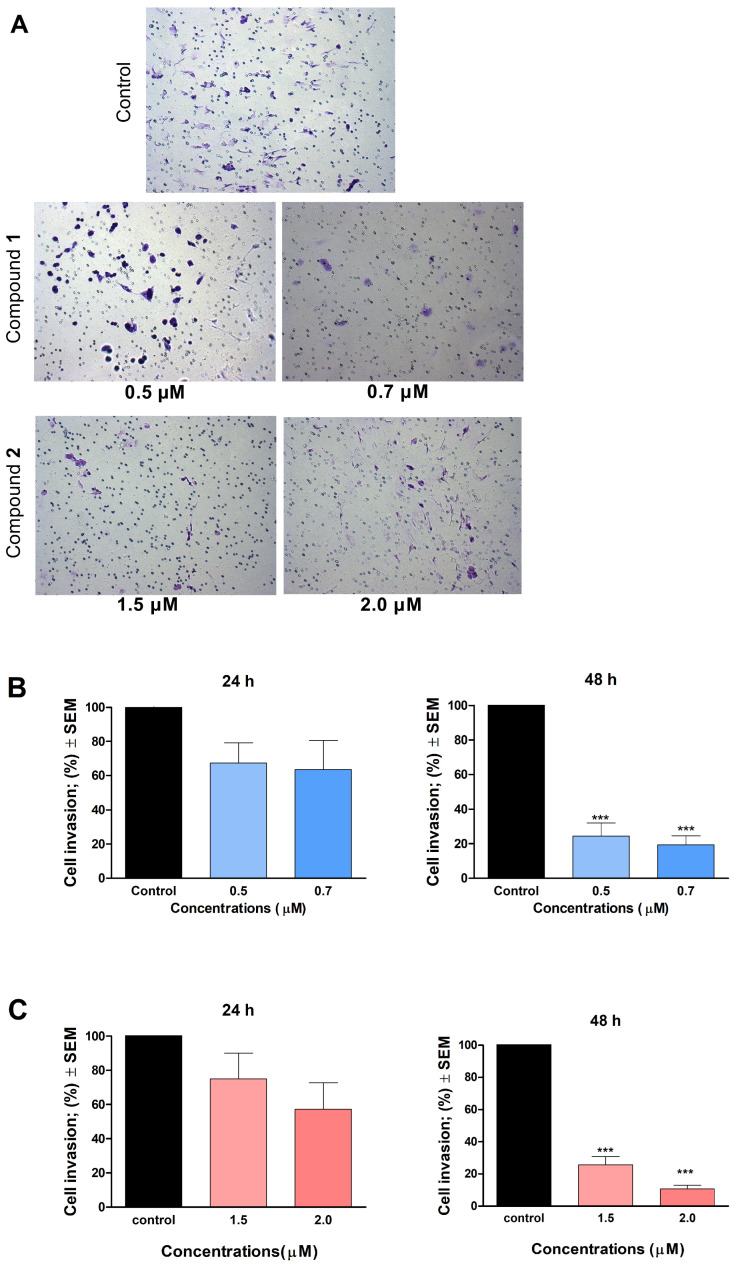
Effect of compounds **1** and **2** on the invasion of A2780 cells. (**A**): representative images at 48 h post-treatment with compounds **1** and **2**. (**B**): calculated number of invading cells treated with **1**. (**C**): calculated number of invading cells treated with compound **2**. *** indicates significance at *p* < 0.001. Findings are based on the results of at least 4 independent experiments, all performed in duplicate. Non-significant changes are not shown.

In addition to cell migration, cellular invasion and infiltration of the surrounding tissues are crucial to the development of tumor metastases. Therefore, we supplemented our wound healing assay of cellular migration with a particular Boyden chamber assay, which models the extracellular environment of primary tumors to study cellular invasion. Both compounds **1** and **2** elicited a modest action on the invasion of A2780 cells after 24 h of incubation (Figure 8). In contrast, a more prolonged incubation period (48 h) revealed impressive results for both compounds.

## 3. Discussion

In recent decades, considerable attention was paid to monoterpenes exerting promising anticancer activity, and numerous studies were conducted to investigate their antiproliferative and antimetastatic properties. Consequently, innumerable terpenoids with anticancer potential were described recently [17,18,19]. The importance of natural compounds and their derivatives in the research and development of anticancer agents is continuously increasing. In a previous study, we investigated some newly synthesized monoterpene-based 2,4-diaminopyrimidine derivatives, and concluded that two of them exhibited remarkable antiproliferative activities [16].

Our present study aimed to assess the antitumor and antimetastatic properties of these potent analogs, as well as to elucidate the mechanism of their action. Antiproliferative assays were performed with these two monoterpene-based 2,4-diaminopyrimidine type derivatives (**1** and **2**) on a panel of human adherent cell lines (Hela, SiHa, MCF-7, MDA-MB-231, and A2780) to calculate IC_50_ values. Based on these results, the anticancer effect of compounds **1** and **2** was selective for A2780 cells (calculated IC_50_ values: 0.76–2.82 μM). Moreover, under the experimental conditions used, the potency of our test compounds was found to be comparable to that of cisplatin, a standard drug routinely used in the therapy of ovarian carcinomas [20]. Tumor specificity is one of the greatest challenges new anticancer drug candidates tend to face. These potent analogs exhibited higher IC_50_ values on NIH/3TE non-cancerous fibroblasts than on ovary cancer cells, indicating high tumor selectivity.

In addition to cancer selectivity, apoptosis induction is a critical feature expected from lead compounds to be suitable for further development as an anticancer agent. Several plant-derived natural products, capable to induce apoptosis, were tested in clinical trials. In general, apoptosis plays a crucial role in controlling and possibly terminating the uncontrolled growth of cancer cells. Utilizing the cellular mechanisms that induce cell death is an exploitable and practical approach in cancer therapy [21,22]. The apoptosis-inducing properties of our currently investigated analogs (**1** and **2**) were demonstrated by fluorescent double staining and cell cycle analysis. The early process of apoptosis is characterized by morphological changes at the cellular level, including cell shrinkage, chromatin condensation, and loss of membrane integrity [21]. These phenomena were visualized by Hoechst 33,258 and propidium iodide fluorescent staining, which was applied on A2780 cells after 24 h and 48 h of incubation with different concentrations of **1** and **2**. Substantial and statistically significant increases in the proportion of apoptotic and necrotic cell populations were observed in A2780 samples treated with either of the test compounds, indicating their possible proapoptotic properties. Furthermore, increasing the concentration of the test compounds led to the appearance of late apoptotic or secondary necrotic cells, showing a loss of membrane function visualized by propidium iodide.

To assess the mechanism of action of **1** and **2**, cell cycle analyses were carried out by flow cytometry on the A2780 cell line. Both test compounds induced a concentration-dependent disturbance of cell cycle. In general, the most noticeable consequence of treatment was the elevation of G2/M populations and the accumulation of hypodiploid (subG1) cells at the expense of the G1 population. Furthermore, the increase in the G2/M population was typically more substantial after 48 h than after 24 h of incubation, indicating cell cycle arrest at the G2/M phase. In addition, the proportion of the hypodiploid subG1 fraction, regarded as the apoptotic cell population, increased in a concentration-dependent manner, supporting the proapoptotic property of **1** and **2** in the A2780 cell line. This finding aligns with recent studies showing that terpenoids typically cause G2/M arrest of the cell cycle and apoptotic cell death in different human cancer cell lines [23,24].

Several studies revealed that some terpenoids induced pronounced cell cycle arrest via direct influence on tubulin polymerization during mitosis [25,26,27]. Tubulin polymerization is a well-organized and crucial process of mitotic cell division. It is generally accepted that tubulin-binding agents induce cell cycle arrest in the G2/M phase via the disruption of microtubule dynamics. Since **1** and **2** was detected to arrest cell cycle in the G2/M phase, it was rational to investigate the direct effect of these analogs on tubulin polymerization in an in vitro cell-free system. Both compounds, and especially compound **2** at 300 μM, increased the maximum rate of microtubule formation and enhanced tubulin polymerization, as reflected by significantly increased Vmax values. Consequently, imbalance between the polymerization and depolymerization of microtubules occurred, leading to cell cycle arrest and induced apoptosis. These findings suggest that our test compounds may have a paclitaxel-like microtubule-stabilizing effect.

Previously, numerous papers reported that ovarian carcinoma has the highest case-to-fatality ratio among all gynecologic malignancies. It is characterized by rapid growth, early metastasis development, and a generally aggressive disease course. Having no early signs and symptoms that may warm for the diagnosis, the high mortality of ovarian cancer is mainly attributable to the fact that most patients present at an advanced stage, with extensive metastatic disease [28]. During tumor progression, including invasive growth and metastasis development, cell invasion is crucial for cancer spread and for the appearance of distant metastases. To achieve reduction in the mortality of ovarian carcinoma, research for novel drug candidates capable of interfering with metastasis formation is eagerly required. The metastatic cascade represents a multi-step process, involving cancer cell migration and invasion through the basement membrane to reach the extracellular matrix and neighboring tissues [29]. In our study, compounds **1** and **2** significantly inhibited the invasion and migration of A2780 cells after 48 h of exposure. We also demonstrated that the inhibition of cell motility becomes apparent at 0.5 μM of **1** and 1.5 μM of **2**, which are below the concentration of substantial antiproliferative activity. Our findings indicate that besides their antiproliferative, proapoptotic, and tubulin-disrupting activities, compounds **1** and **2** also can be potent antimigratory agents because of their significant inhibitory effects on cell migration and invasion. Our study is the first one to demonstrate the mechanism of action of monoterpene-based 2,4-diaminopyrimidine derivatives on tumor cells, clarifying our understanding of how they influence cell cycle, apoptosis, and motility of cancer cells.

In conclusion, our results demonstrated that compounds **1** and **2** are promising antiproliferative agents in vitro; both cause G2/M phase disturbance during the cell cycle presumably by stabilizing tubulin polymers, leading to programmed cell death. In addition to their proapoptotic effect, they are suggested to show antimetastatic potential in the utilized ovarian cancer cell line. Based on our findings presented above, the tested compounds can be regarded as potential new drug candidates with a promising new mechanism of action among terpenoids possessing antiproliferative properties, and they may be utilized for the design of novel anticancer agents.

## 4. Materials and Methods

### 4.1. Chemicals

A series of novel monoterpene-based 2,4-diaminopyrimidine type derivatives (**1** and **2**, Figure 1) were synthesized by Raji et al. as described previously [16]. For all in vitro experiments, stock solutions (10 mM) of the test compounds were prepared with dimethylsulfoxide (DMSO).

### 4.2. Cell Cultures

The cancer cell lines utilized for our investigations, including human ovarian carcinoma (A2780), human cervix carcinoma (Hela), human breast adenocarcinomas (MCF-7 and MDA-MB-231), and mouse embryonic fibroblast cell line (NIH/3T3) were purchased from the European Collection of Authenticated Cell Cultures (ECACC, Salisbury, UK). Another cervical cell line (SiHa) was acquired from the American Tissue Culture Collection (Manassas, VA, USA). For optimal growth, all cell lines were maintained in minimal essential medium (MEM) supplemented with 10% heat-inactivated fetal bovine serum (FBS), 1% MEM non-essential amino acid solution (MEM-NEAA), and 1% penicillin/streptomycin mixture, at 37 °C, in a humidified incubator, containing 5 % carbon dioxide (CO_2_). All the utilized media and supplements were purchased from Lonza Group Ltd. (Basel, Switzerland). Unless otherwise specified, the chemicals and kits used for the experiments were purchased from Merck Life Science Ltd. (Budapest, Hungary).

### 4.3. Antiproliferative (MTT) Assay

To assess the antiproliferative properties of monoterpene-based 2,4-diaminopyrimidine type derivatives, a standard MTT assay was performed [30]. Cells were seeded onto 96-well microplates at a density of 5000 cells/well. After incubation overnight, cells were treated with increasing concentrations of the test compounds (0.1–30 µM), and they were incubated for 72 hours under cell culture conditions as noted above. Subsequently, 3-(4,5-dimethylthiazol-2-yl)-2,5-diphenyltetrazolium bromide solution (MTT, 5 mg/mL in phosphate buffer) was added to the wells, followed by incubation for 4 h. The supernatant was removed, the precipitated formazan crystals were then dissolved in 100 μL DMSO. Finally, absorbance values were recorded by a microplate reader (BMG Labtech, Ortenberg, Germany) at 545 nm. Untreated cells were used as a control, and cisplatin was used as a clinically applied reference agent. Six-point dose–response curves were fitted to the measured points, and the IC_50_ values were calculated by the GraphPad Prism 5.0 software (GraphPad Software, San Diego, CA, USA). Two independent measurements were performed with five parallel wells.

### 4.4. Hoechst 33258–Propidium Iodide Fluorescent Double Staining

Fluorescent double staining was performed to characterize the changes in cell morphology and cell membrane integrity after treatment with the test compounds. Firstly, A2780 cells were seeded onto 6-well plates at a density of 300,000 cells/well, and were incubated under cell culture conditions overnight before being treated with different concentrations of the test compounds for 24 h and 48 h. Next, the cells were stained by lipophilic Hoechst 33,258 (1 μg/mL, HO) and hydrophilic propidium iodide (3 μg/mL, PI) for one h under the same circumstances described previously. After the incubation period, the medium in the wells was refreshed. Then, images were taken by a Nikon Eclipse TS100 microscope (Nikon Instruments Europe, Amstelveen, The Netherlands) equipped with appropriate optical blocks. The images were analyzed by the NIS-Elements BR software (Nikon Instruments Europe, Amstelveen, The Netherlands). Nuclei emitting fluorescence values were counted, and the proportions of intact, apoptotic, and necrotic cell populations were expressed as percentages.

### 4.5. Cell Cycle Analysis

Cell cycle analysis was performed on ovarian cancer cell line A2780 to elucidate the exact mechanism of action of the test compounds [31]. Briefly, cells were seeded onto 24-well plates (80,000 cells/well). After incubation overnight, the samples were treated with increasing concentrations of the test compounds for 24 h and 48 h. Samples were collected after a washing step with phosphate-buffered saline (PBS) and the trypsinization process. Cells were pooled with the collected supernatants and were centrifuged at 1400 rpm for 5 min at room temperature. Pellets were resuspended and stained by PI solution, which contained 10 µg/mL RNase A and 0.1% sodium citrate dissolved in PBS, for 30 min in the dark, at room temperature. A FACSCalibur flow cytometer was used to detect DNA content of at least 20,000 cells/sample, and the recorded data were analyzed by the Kaluza Analysis Software (Beckman Coulter, Brea, CA, USA). Untreated cells were considered as control. Hypodiploid subG1 phases were regarded as a sign of a late apoptotic event [32].

### 4.6. Tubulin Polymerization

An in vitro tubulin polymerization assay was performed to determine the direct effect of the test compounds on the microtubular system (Cytoskeleton Inc., Denver, CO, USA) [33]. First, the assay reactions were performed on a pre-warmed (37 °C), UV-transparent, 96-well microplate. Then, 10 µL of 175 µM and 350 µM solution of test compound **1**, as well as 10 µL of 150 µM and 300 µM solution of test compound **2**, was added to the wells supplemented with 2 mM MgCl_2_, 0.5 mM ethylene glycol tetraacetic acid (EGTA), 1 mM guanosine triphosphate (GTP) and 10.2% glycerol. Ten microliters of general tubulin buffer were used as an untreated control, and paclitaxel (PAC) served as the reference agent. The polymerization reaction was initiated by adding 100 μL of 3.0 mg/mL tubulin in 80 mM PIPES, pH 6.9, to each sample. Absorbance of the samples was recorded per minute by a microplate reader (BMG Labtech, Ortenberg, Germany) at 340 nm, and a 60 min kinetic measurement protocol was applied. The maximum reaction rate (Vmax; Δabsorbance/min) of the test compounds was calculated from the highest difference between the measured absorbances at two consecutive time points. According to the manufacturer’s recommendation, a clinically applied reference agent, paclitaxel (PAC), was used at a high concentration (10 μM). Each sample was prepared in two parallels.

### 4.7. Wound-Healing Assay

A wound-healing assay was carried out to determine the test compounds’ effect on cell migration, using the ibidi Culture-Insert with two wells separated by a special silicone insert (Ibidi GmbH, Gräfelfing, Germany) [34]. A2780 cell suspension was prepared in MEM, supplemented with 2% FBS, 1% MEM-NEAA, and 1% penicillin/streptomycin mixture. Firstly, cells were seeded onto both chambers at a density of 50,0000 cells, then were incubated for 48 h until a confluent monolayer was formed. Then, the insert was gently removed, and a washing step was implemented to remove non-adherent cells. Next, the test compounds were dissolved in a medium containing 2% FBS, and the desired concentrations of the solutions (either compound **1** or **2**) were added to the cells, followed by incubation for 24 h and 48 h. Images of the wound area at different time points (0, 24, and 48 h) were taken with a Nikon Eclipse TS100 fluorescence microscope. The size of the cell-free area was determined manually along the wound edge by the ImageJ software (National Institutes of Health, Bethesda, MD, USA). Cell migration rate was calculated by comparing wound closure in treated wells relative to untreated controls.

### 4.8. Boyden Chamber Assay

The Boyden chamber assay, regarded as one of the most widely accepted cell migration techniques [35], was used to investigate the invasion ability of A2780 cells treated with the test compounds. It was performed using a BD BioCoat Matrigel Invasion Chamber (BD Biosciences, Bedford, MA, USA), containing a polyethylene terephthalate (PET) membrane of 8 μm pore size and a thin layer of matrigel basement matrix as an in vitro model of the extracellular environment. Suspension of the A2780 cells, prepared in serum-free medium supplemented with sub-antiproliferative concentrations of the test compounds was injected into the upper chamber, right onto the prehydrated membrane. A medium supplemented with 10% FBS was used as a chemoattractant in the lower chamber. After 24  h and 48 h of incubation, the supernatants and non-invading cells were removed cautiously with a cotton swab, and the membranes were washed with PBS twice. Samples were fixed in ice-cold 96% ethanol for 15 min, they were then rewashed with PBS and were stained with 1% crystal violet dye for 30 minutes in dark, at room temperature. A Nikon Eclipse TS100 microscope was utilized to take images (at least 3 per insert). Invading cells were counted to determine invasion rate, and treated samples were compared to untreated controls.

### 4.9. Statistical Analysis

For each experiment, GraphPad Prism 5.01 (GraphPad, San Diego, CA, USA) was used for the statistical evaluation of experimental data. One-way analysis of variance (ANOVA) with Dunnett post-test was applied to estimate statistical significance. A *p*-value < 0.05 was regarded as statistically significant.

## Figures and Tables

**Table 1:** Antiproliferative properties of compounds **1** and **2**.

Cell Line	Calculated IC_50_ Values (µM) (95% Confidence Interval (µM))		
	Compound 1	Compound 2	Cisplatin
HeLa	1.39	7.69	12.14
	(1.28–1.53)	(6.61–8.93)	(10.18–14.46)
SiHa	2.14	16.70	5.31
	(1.74–2.64)	(12.72–21.92)	(4.65–6.05)
MDA-MB-231	1.78	27.35	10.17
	(1.46–2.17)	(22.95–32.61)	(8.01–12.92)
MCF-7	1.49	16.27	8.05
	(1.26–1.77)	(1.07–2.47)	(6.24–10.40)
A2780	0.77	2.86	1.37
	(0.69–0.88)	(2.34–3.49)	(1.25–1.50)
NIH/3T3	4.36	8.05	3.96
	(3.31–5.74)	(5.17–12.54)	(3.37–4.66)

## Data Availability

Data are available upon request.

## Supplementary Materials

The following supporting information can be downloaded at: https://www.mdpi.com/article/10.3390/ijms241310581/s1, Figures S1–S5: Representative microscopic images of A2780 cells treated with compounds **1** or **2**.
