# Supplementary material for: Investigation of Anticancer Properties of Monoterpene-Aminopyrimidine Hybrids on A2780 Ovarian Cancer Cells
Source: Int J Mol Sci. 2023 Jun 24;24(13):10581. doi: 10.3390/ijms241310581 (PMC10341452; doi:10.3390/ijms241310581)
Supplement: Supplementary file 1 [file ijms-24-10581-s001.zip › ijms-2430599-supplementary.pdf]

Supporting information

Investigation of anticancer properties of monoterpene-aminopyrimidine hybrids on A2780 ovarian cancer cells

Viktória Nagy, Raji Mounir, Gábor J. Szebeni, Zsolt Szakonyi, Nikolett Gémes, Renáta Minorics, Péter Germán and István Zupkó

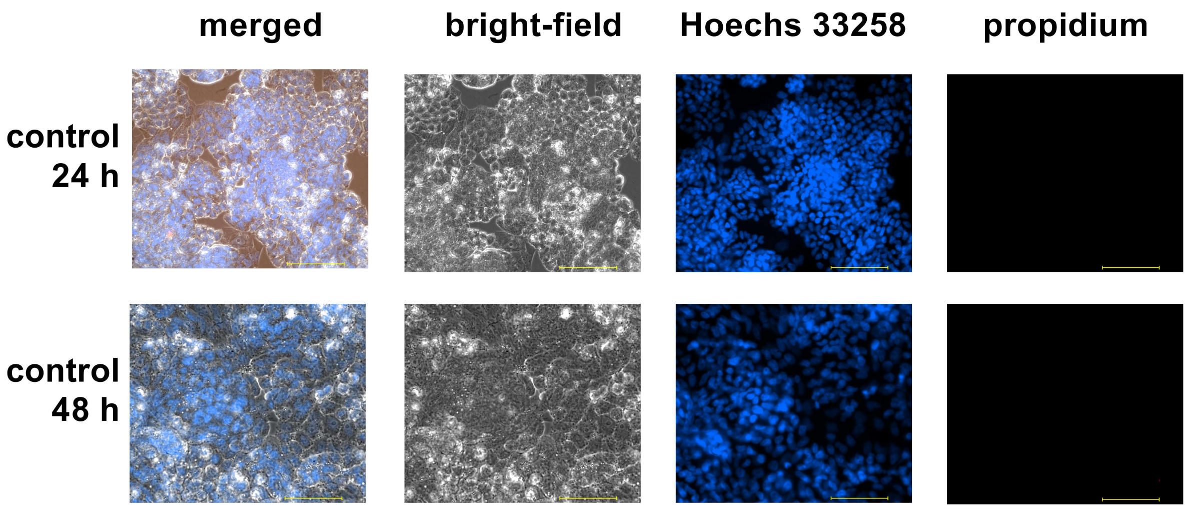

Figure S1. Control (untreated) A2780 cells after 24 or 48 h incubation. The bar in the pictures indicates 100 μm.

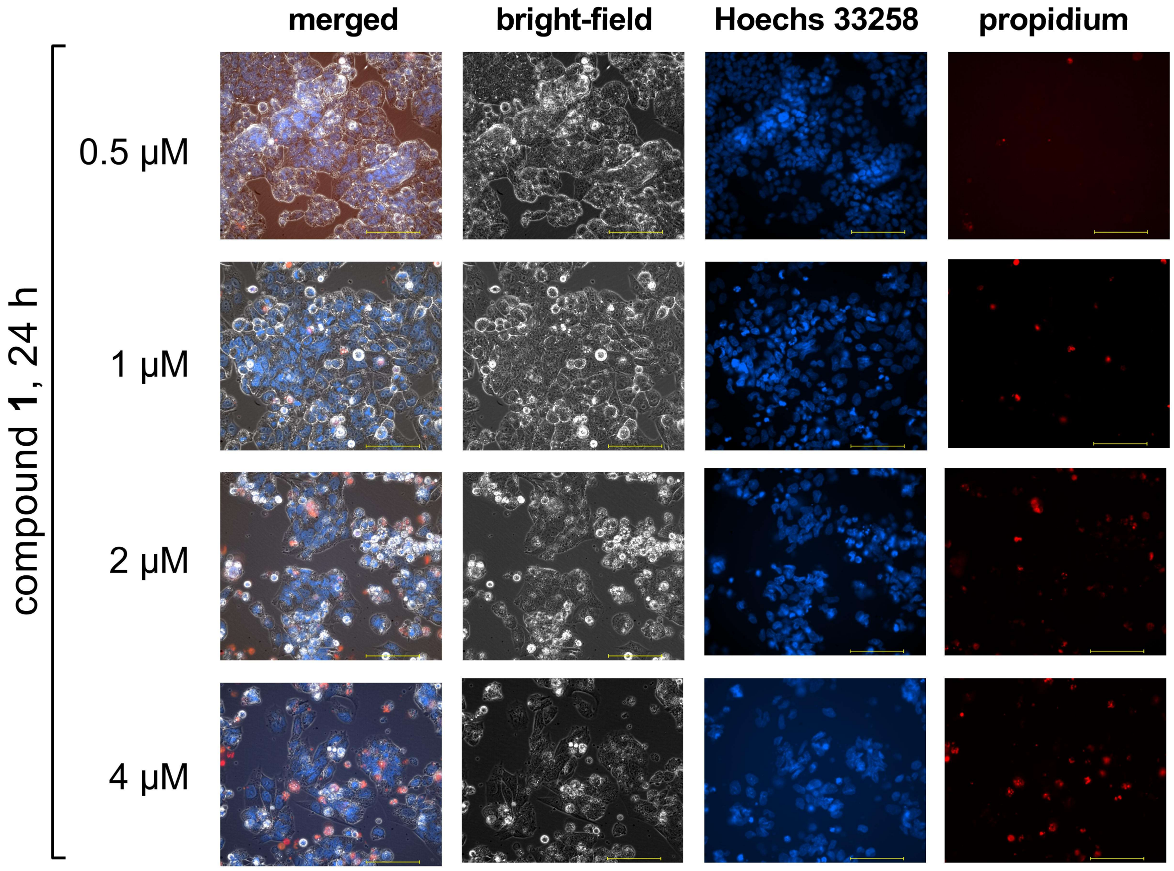

Figure S2. A2780 cells treated with compound **1** for 24 h. The bar in the pictures indicates 100 µm.

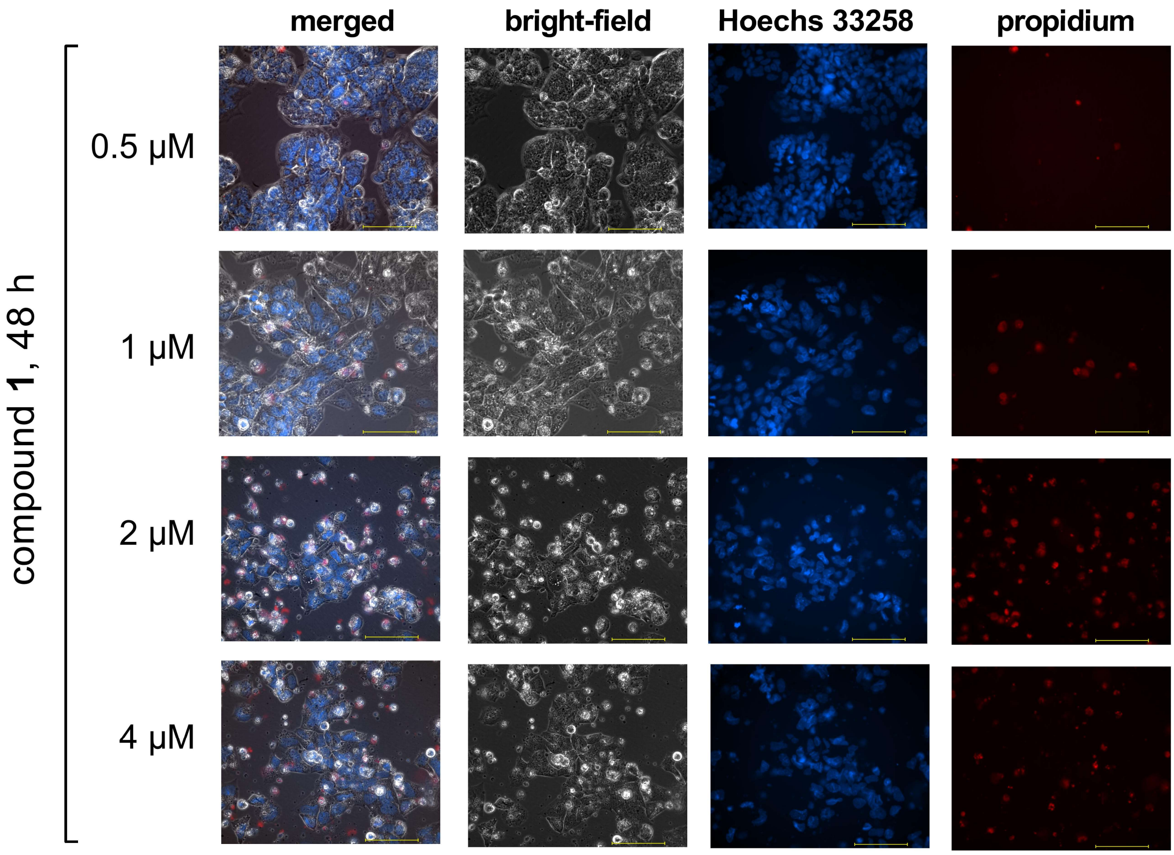

Figure S3. A2780 cells treated with compound **1** for 48 h. The bar in the pictures indicates 100 μm.

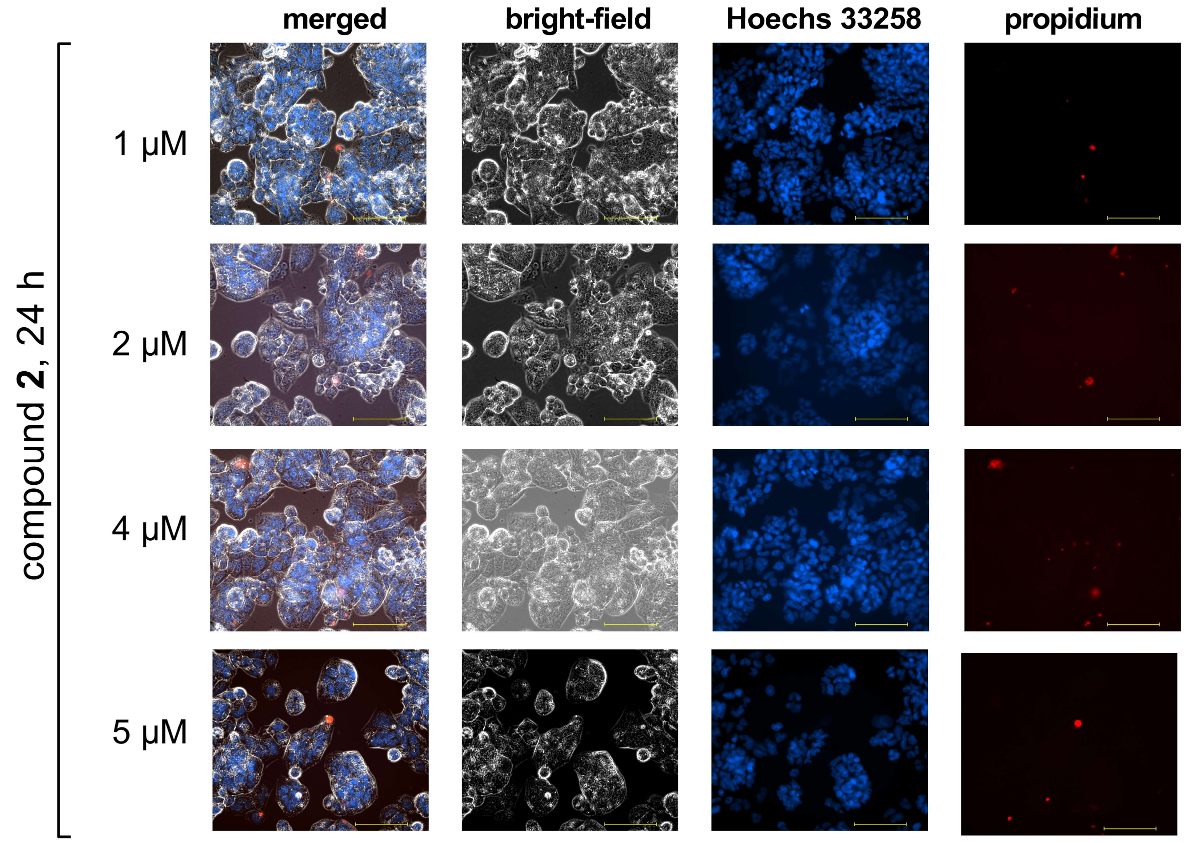

Figure S4. A2780 cells treated with compound **2** for 24 h. The bar in the pictures indicates 100 µm.

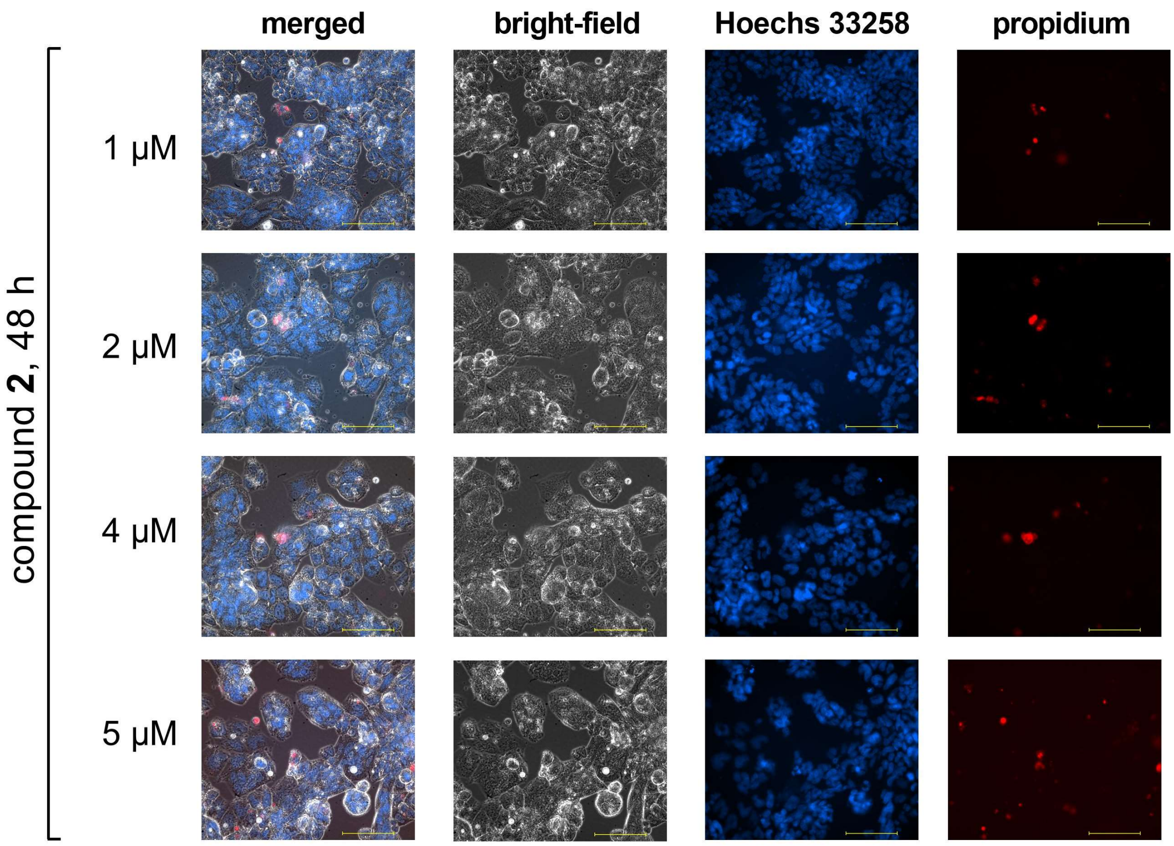

Figure S5. A2780 cells treated with compound **2** for 48 h. The bar in the pictures indicates 100 μm.
